# Effects of the Combined PDL/Nd:YAG Laser on Surgical Scars: Vascularity and Collagen Changes Evaluated by *In Vivo* Confocal Microscopy

**DOI:** 10.1155/2014/204532

**Published:** 2014-09-09

**Authors:** Krisztina Vas, Magdolna Gaál, Erika Varga, Réka Kovács, Balázs Bende, Ádám Kocsis, Lajos Kemény

**Affiliations:** ^1^Department of Dermatology and Allergology Clinic, University of Szeged, Korányi Fasor 6, Szeged 6720, Hungary; ^2^MTA-SZTE Dermatological Research Group, Korányi Fasor 6, Szeged 6720, Hungary

## Abstract

The aim of this study was to investigate the efficacy of the sequential combined 585 nm PDL and the 1064 nm neodymium:yttrium-aluminium-garnet laser (PDL/Nd:YAG) in the treatment of surgical scars and to evaluate the short-term effects by* in vivo* confocal microscopy (RCM) and the long-term effects by clinical assessment of the scars. Twenty-five patients were enrolled with 39 postoperative linear scars; each scar was divided into two fields. One half was treated with the combined PDL/Nd:YAG laser, whereas the other half remained untreated. Each scar was treated three times at monthly intervals. Scars were evaluated by an independent examiner, using the Vancouver Scar Scale. The combined PDL/Nd:YAG laser significantly improved the appearance of the scars. In order to study the short-term effects of combined laser treatment, six additional patients were enrolled with 7 postoperative linear scars. One half of scars was treated once with the combined PDL/Nd:YAG laser. One week after this laser treatment, both the treated and the nontreated parts of the scars were examined by dermoscopy and RCM. The dermoscopic pictures revealed improvements even in treated areas. In conclusion, the combined PDL/Nd:YAG laser was found to be effective in improving the quality and appearance of the surgical scars.

## 1. Introduction

Despite modern suturing techniques, operations may be followed by the development of nonesthetic scars. Surgical scars often remain red in color, firm, and elevated to the touch. Some scars may persist in a hyperpigmented form or may become hypertrophic or keloid. These nonesthetic scars may come to be regarded as permanent stigmas and often cause considerable esthetic and psychological problems for patients. Various therapeutic modalities have been attempted with the aim of improving the clinical appearance of scars, with differing degrees of success, for example, corticosteroid injection, dermabrasion, surgical revision, chemical peeling, silicone gel application, vitamin E-based remedies, pressure therapy and X-ray irradiation, cryosurgery, 5-fluorouracil (a pyrimidine analog), and certain antitumor agents (e.g., Bleomycin) [1–6].

Lasers have also been applied to improve the appearance of such scars. The first lasers utilized for this purpose were ablative, nonselective lasers: carbon dioxide (CO_2_) or erbium:yttrium-aluminium-garnet (Er:YAG) laser [7–11].

Nouri et al. recently studied the use of the pulsed dye laser (PDL) at different wavelengths (585 nm and 595 nm) and with different pulse durations (short and long), and Conologue and Norwood demonstrated that the cryogen-cooled 595 nm PDL treatment is effective in improving the wound healing [12–15].

Investigations of the therapeutic efficacy of different laser and light source combinations (e.g., the combination of the PDL and the Nd:YAG laser) are important in view of their complementary nature. The skin contains a number of light-absorbing substances (chromophores) such as water, melanin, hemoglobin, and exogenous pigments. An initial low PDL pulse selectively increases the local blood temperature to 62–80°C, resulting in oxyhemoglobin-methemoglobin conversion. A recent study revealed that the light of the Nd:YAG laser is preferentially absorbed by methemoglobin rather than by oxyhemoglobin. The high temperature attained induces blood coagulation in the vessels of the treated area. The combination of these lasers functions via selective photothermolysis, which targets the blood vessels with minimal damage to the surrounding tissues.

As sequential combined PDL/Nd:YAG laser treatment has been shown to be highly effective for the treatment of acne vulgaris, cutaneous photoaging, and leg veins, the aim of our present study was to investigate the efficacy of combined PDL/Nd:YAG laser treatment on the appearance of scars, when used immediately after suture removal [16–18].

In this study, treatment was performed on regular scars after surgery starting right after the removal of sutures. Our results conclude that combined PDL/Nd:YAG laser therapy starting on the day of suture removal is safe and effective in improving the quality and cosmetic appearance of surgical scars in patients with skin types I–IV; the improvement was confirmed at structural level by dermoscopy,* in vivo* confocal microscopy, and VSS. RCM permits the real-time imaging of human skin including various skin disorders. It is a noninvasive high-resolution imaging technique which allows the structural and cellular visualization and analysis of the skin to a depth up to 250 *μ*m. This technique can be used in a wide range of dermatological settings. Objective data can be obtained on the skin structure and its alterations in various conditions. It is also widely used to evaluate almost any kind of superficial skin lesions. The RCM assist of the diagnosis and differential diagnosis of skin diseases can be refined and a more specific opinion can be provided without or before a surgical biopsy. It can help in the assessment of lesions with a clinical and dermoscopic suspicion of malignancy eliminating the performance of unnecessary surgical biopsies or confirming a previous diagnosis in order to plan the optimal surgical solution [19–21].

## 2. Methods

### 2.1. Effects of the Combined PDL/Nd:YAG Laser on the Clinical Appearance of the Scars

Twenty-five (22 female and 3 male) participants between the age of 17 and 50 years, with Fitzpatrick skin types I–IV and linear scars greater than 3 cm originating from nevus removal, were enrolled into the study (Table 1). The patients had been operated on at the Plastic Surgery Unit of the Department of Dermatology and Allergology Clinic in Szeged. All of the patients involved in the study had otherwise healthy skin and were not suffering from major medical illnesses. Exclusion criteria were pregnancy, lactation, medicines that increase sensitivity to light, anticoagulant medication, a history of malignancy, and susceptibility to keloids and hypertrophic wound healing. All surgical procedures had been carried out by the same group of plastic surgeons, and wound closure had been performed with standardized suturing techniques (two-layer intracutaneous running, one deeper absorbable suture and one superficial nonabsorbable suture). The sutures were removed 14 days after the operation.

The patient's informed consent was obtained in all cases before laser treatment was started immediately after suture removal, followed by repeat treatments 4 and 8 weeks later.

Twenty-five patients with 39 postoperative linear scars were treated three times at monthly intervals with the combined 585 nm PDL and 1064 nm Nd:YAG laser (Cynergy, Cynosure Inc., Westford, MA, USA).

Each scar was divided into two fields; one half of each scar was treated with the combined PDL/Nd:YAG laser and the other half remained untreated. The treated and the control halves were selected randomly.

The 585 nm PDL was used with a spot size of 10 mm with 10% overlap and a pulse duration of 0.5 ms at a fluence of 4.5 J/cm^2^, in combination with treatment with the 1064 nm Nd:YAG laser with a spot size of 10 mm and a pulse duration of 15 ms at a fluence of 60 J/cm^2^, with 500 ms time delay between the delivery of the two wavelengths. Specific safety eyeglasses were worn during the treatment. The skin surface was cooled with a continuous flow of cold air. The patients reported only minimal pain during treatment; erythema appeared around the scar 1-2 days after the laser treatment. No serious side-effects of the treatment were observed. All photographs were taken by one photographer with a* Nikon D200 *camera, under identical conditions of illumination and patient positioning. Analysis was performed on 39 scars in a blind study by an independent examiner one month after the last treatment (third laser irradiation).

The level of improvement of the scars was evaluated by using the Vancouver Scar Scale (VSS) one month after the final treatment. This scale is the internationally accepted method for the description of scars. The VSS consists of four variables: vascularity, height (thickness), pliability, and pigmentation. Each variable has four to six possible scores. A total score ranges from 0 to 13, where the score of 0 reflects normal skin.

Statistical analysis was performed with the SPSS 15.0 software.

The Wilcoxon test chosen for the evaluation allows determination of the degree of improvement based on the VSS (pigmentation, vascularity, pliability, and height) scores of the scars in both the treated and the untreated parts.

### 2.2. Effects of the Combined PDL/Nd:YAG Laser Treatment on Blood Vessels and on Collagen Fibers

To determine the effects of the combined PDL/Nd:YAG laser therapy on scar microcirculation, dermoscopy and* in vivo* confocal microscopic analyses were performed.

Half of each scar in 6 patients (5 female and 1 male) with 7 postoperative linear scars was treated immediately after suture removal once with the combined PDL/Nd:YAG laser, and, one week later, both parts of the scar were examined by dermoscopy and reflectance-mode* in vivo* confocal microscopy (RCM).

One week after the combined laser treatment, the dermoscopic and RCM images of the 7 scars of the 6 patients were analyzed with the LUCID VivaScope 1500 Multilaser (MAVIG GmbH, Munich, Germany) equipment, using the 785 nm wavelength laser of the machine. Our objectives were to analyze the microcirculation and the structures of the epidermis and collagen bundles and to assess the differences between the treated and untreated areas. Images were made first of the combined PDL/Nd:YAG laser-treated and then of the control areas. The captured areas also contained the neighboring normal skin. The first step was to obtain a dermoscopic picture of the examined area with the RCM machine camera. The zero level was then determined and this level was marked relative to the surface of the scar tissue. The first image was intended to be taken at the level of the stratum granulosum-spinosum. We captured the stratum corneum of the scar as the zero level and then defined the first level which was usually 25–30 *μ*m deeper than the zero one. Three further images were next obtained, each 25 *μ*m deeper than the previous one. Due to the above mentioned surface irregularity, we often had to make an additional fifth layer to visualize the deeper structures appropriately. The maximum area of 8 × 8 mm was captured at each level. We also made two Vivastack captures at representative parts of the scar and a comparative one at the normal skin area. These Vivastack images were taken from the level of the stratum granulosum-spinosum down to 150 *μ*m in 1.5-*μ*m steps. Vessels were detected and compared on the basis of the dermoscopy and RCM images.

## 3. Results

### 3.1. Effects of the Combined PDL/Nd:YAG Laser on the Clinical Appearance of the Scars

**Figure 1:**
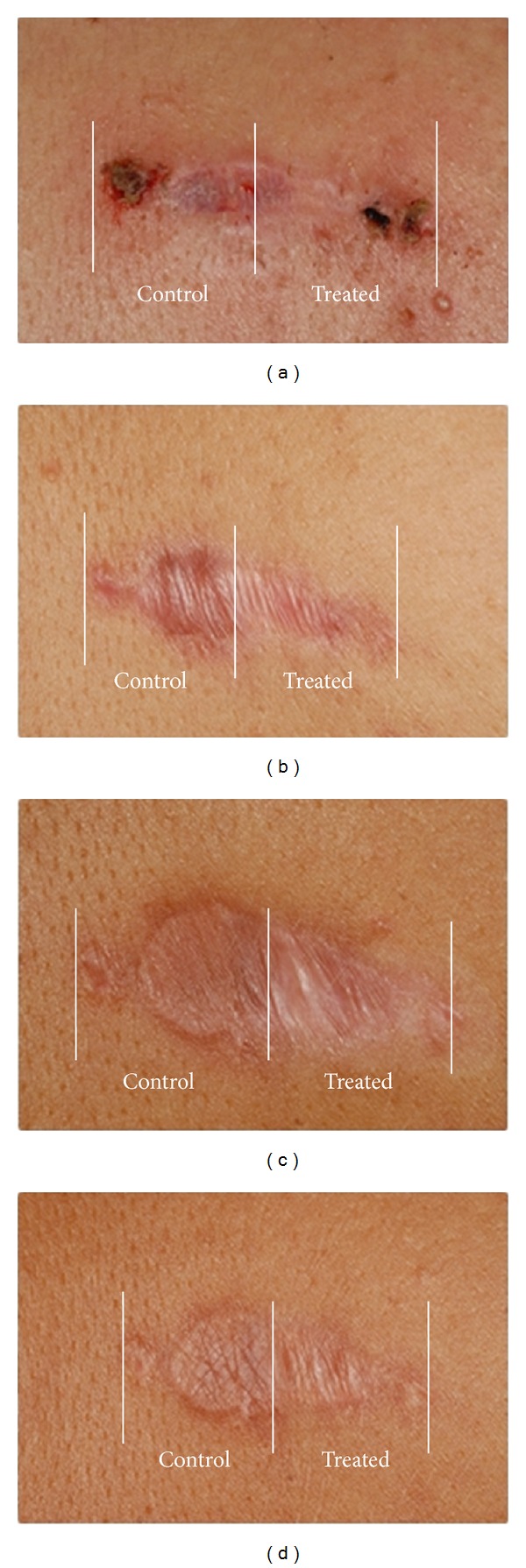
Effects of combined 585/1064 nm laser treatment on the clinical appearance of the scar back of in the patient number 16 (Fitzpatrick skin type IV) on the day of suture removal (a), 1 month after the first treatment (b), 1 month after the second treatment (c), and at the final assessment (d). The treated half (Treated) and the control half (Control) of the scar are indicated.

**Figure 2:**
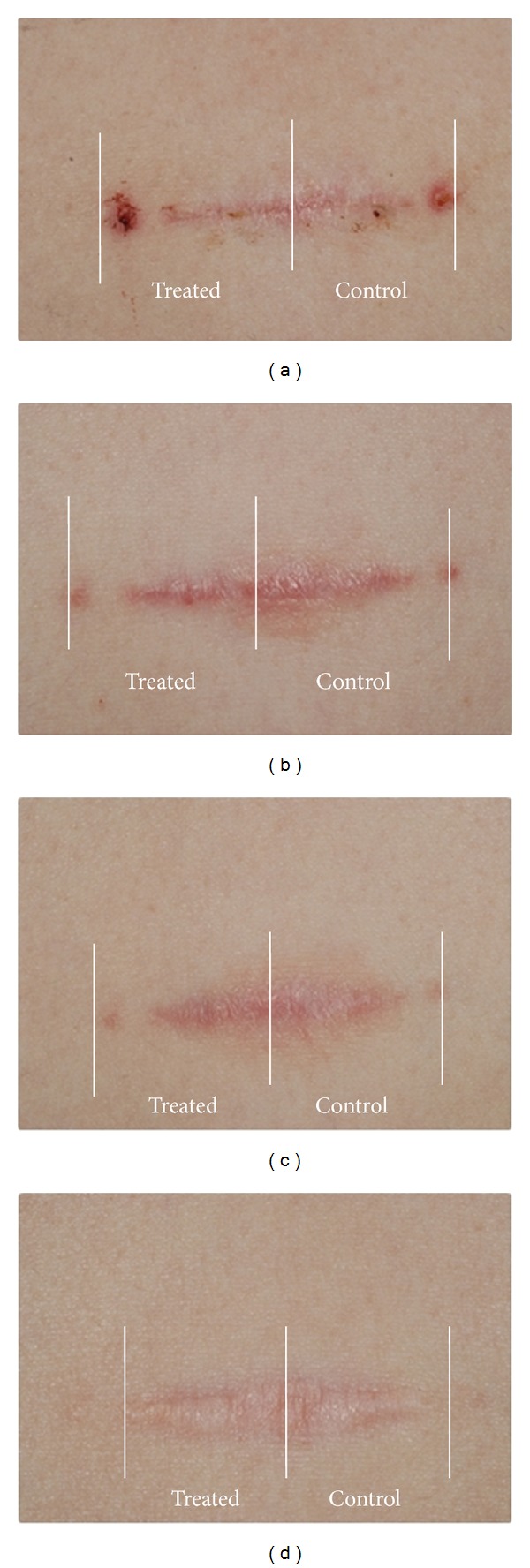
Effects of combined 585/1064 nm laser treatment on the clinical appearance of the scar waist of in the patient number 10 (Fitzpatrick skin types I) on the day of suture removal (a), 1 month after the first treatment (b), 1 month after the second treatment (c), and at the final assessment (d). The treated half (Treated) and the control half (Control) of the scar are indicated.

The combined PDL/Nd:YAG laser treatment resulted in a significant clinical improvement of the scars (Figures 1 and 2). The surgical scar is depicted from the day of suture removal to the final combined laser treatment. One month after the third combined laser treatment, the final analysis revealed a significant difference in favor of the treated parts of the scar.

**Figure 3:**
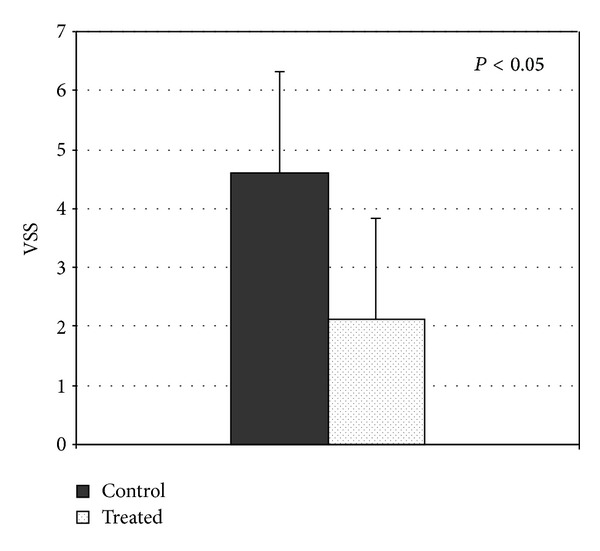
VSS score 1 month after final treatment and final evaluation (*n* = 25); *P* < 0.05 was regarded as a significant change.

The VSS parameters (*n* = 39 scars) demonstrated a significant dermoscopic improvement relative to the control (Figure 3). A worsening status of wound healing after laser treatment was detected only in one patient, where we observed pink vascularity, hyperpigmentation, supple, and 2–5 mm heightened parts of the scar at the treated region.

**Figure 4:**
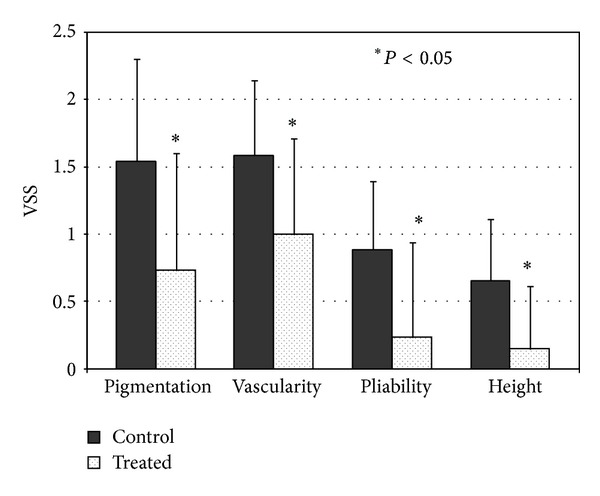
Improvement in individual VSS parameters 1 month after the final combined PDL/Nd:YAG laser treatment (*n* = 25); *P < 0.05 was regarded as a significant change.

Each of the four parameters (pigmentation, vascularity, pliability, and height) was improved significantly 1 month after the final combined laser treatment (Figure 4). There were no significant complications during this study. Both patients and doctors were satisfied with the cosmetic appearance of the surgical scars after final combined laser therapy. Three months after the last laser treatment, further improvement in the clinical appearance of the laser treated scars was observed (not shown).

### 3.2. Effects of the Combined PDL/Nd:YAG Laser Treatment on Blood Vessels and on Collagen Fibers

Concerning each of the investigated parameters, the combined PDL/Nd:YAG laser significantly improved the appearance of the scars; thus, we set out to enroll further 6 subjects in order to study the short-term effect of combined laser treatment.

**Figure 5:**
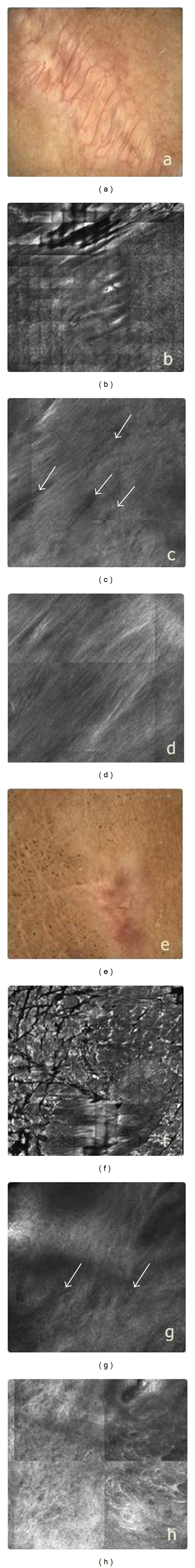
Effects of a single combined 585/1064 nm laser treatment on the blood vessels and collagen fibers (abdomen). The untreated areas ((a), (b), (c), and (d)) and treated areas ((e), (f), (g), and (h)) are indicated. Control area: the dermatoscopic picture revealed high vascularity in the scar tissue ((a) Bar = 92.96 *μ*m). The RCM picture of the scar and its surroundings indicated that the scar is only slightly depressed ((b) Bar = 132.59 *μ*m); there are numerous, parallel collagen fibers with numerous dilated vessels (arrows) among them (c) and numerous parallel collagen fibers ((d) Bar = 132.59 *μ*m). Treated area: the dermatoscopic picture shows low vascularity in the scar tissue (e). The RCM picture of the scar and its surroundings demonstrated that the scar is depressed relative to the normal skin ((f) Bar = 70.10 *μ*m); there were collagen fibers regular and elongated and a few narrow vessels (arrows) among them ((g) Bar = 138.68 *μ*m) and regular and elongated collagen fibers ((h) Bar = 129.54 *μ*m).

One week after the combined PDL/Nd:YAG laser treatment, the dermoscopic pictures of the control, untreated area displayed marked parallel or lace-like vascularity (Figure 5), which was pronounced not only in the scar area but also around it. In several cases, the scar appeared to be slightly elevated; however, more often a depressed characteristic was detectable.

The epidermis exhibited a regular or broadened honeycomb pattern according to the RCM investigation, although some areas revealed atypical keratinocytes as well. The dermoepidermal junction showed nonspecific pattern without papillary contours. Parallel, rough, coarse collagen bundles were detected in the upper dermis, whereas the vessels were dilated and horizontal.

The treated area of the dermoscopic pictures revealed a narrower scar with notably decreased vascularity. The vessels were more delicate and situated apparently haphazardly compared to the untreated part. The treated scars were usually depressed. In some cases, the surroundings of the scar displayed more vascularity than the scar itself. The epidermis also had a broadened honeycomb pattern with a low number of atypical keratinocytes and some dendritic inflammatory cells. At the dermoepidermal junction, the pattern was nonspecific, without papillary contours similar to the untreated area. The collagen fibers were comparably coarse; however, they were not numerous and their orientation was not uniformly parallel. Only low numbers of narrow horizontal vessels were observed.

## 4. Discussion

Different techniques are used to enhance wound healing, but, thanks to the rapid advances in laser technology, a new method has emerged with a promising future for modern wound healing.

The flashlamp-pumped PDL, which emits light at a wavelength of 585 nm, has become the gold standard in the treatment of port-wine stains and also an effective treatment modality for superficial vascular lesions, including those associated with photoaging, such as facial telangiectasias. The Nd:YAG laser emits light at a wavelength of 1064 nm, which allows deep penetration into the dermis and vascular specificity due to a broad absorption peak of oxyhemoglobin above 800 nm [17, 18].

PDL and Nd:YAG lasers of different wavelengths have been incorporated in the novel dual laser device built into the same console. Laser and light source combinations are currently being examined for their complementary, additive, or sometimes synergistic action.

The abnormal blood vessels in the scar tissue area are occluded and absorbed, which results in artificial ischemia, inhibiting nutrient supply to the wound. The laser treatment enhances wound contraction, the remodelling of collagen fibers by thermal necrosis, activation of the release of basic fibroblast growth factor, and inhibition of transforming growth factor *β*1 [1, 2]. The combined laser therapy additionally decreases the accumulation of fibroblast cells in the scar tissue and stimulates the production of reticular collagen fibers [10, 11]. Treatment with the combined 585 nm PDL and 1064 nm Nd:YAG laser is noninvasive, minimally uncomfortable, and requires no anesthesia.

Jung et al. compared a PDL and a combined PDL/Nd:YAG laser in the treatment of acne vulgaris and found that the combined PDL/Nd:YAG laser was significantly better than the PDL in reducing noninflammatory acne lesion counts 8 weeks after the treatment; an improvement was also observed in the treatment of inflammatory acne lesions, though it was not statistically significant [16]. Trelles et al. described the efficiency of the PDL/Nd:YAG laser combination in the treatment of leg veins [18]. Berlin et al. treated cutaneous photoaging symptoms with a combined PDL/Nd:YAG laser. Their examinations indicated the greatest improvements in telangiectasias and diffuse erythema, with slightly less change in epidermal pigmentation and lentigines. The combined sequential PDL/Nd:YAG laser can be used safely and effectively in facial photorejuvenation [17].

Nouri et al. compared the effectiveness of the 585 nm versus the 595 nm PDL in the treatment of new surgical scars. Both lasers improved the cosmetic appearance of the scars statistically. However, 585 nm appeared to be the better wavelength, as it substantially normalized the height in a significant number of the scars, in addition to the vascularity and pliability. It also emerged that both short (450 *μ*s) and long (1.5 ms) 585 nm pulses were safe and effective in improving the quality and cosmetic appearance of surgical scars starting on the day of suture removal. No significant difference was detected between the results with the different pulse durations. The cryogen-cooled 585 nm and 595 nm PDL was used with a spot size of 10 mm or 7 mm and a pulse duration of 450 *μ*s or 1.5 ms at a fluence of 3.5 J/cm^2^ to treat surgical scars starting on the day of suture removal [12–14].

In previous publications, the most significant improvements were found in vascularity and pliability after therapy with the 585 nm or 595 nm PDL [12–15].

Bencini et al. have described the long-term effects of carbon dioxide (CO_2_) laser treatment on skin aging after fractional laser therapy lasting 6 weeks to 3 months at least. Epidermal changes and collagen remodeling were evaluated by* in vivo* confocal microscopy [22]. Recently, Sattler et al. treated healthy probands with fractional carbon dioxide laser; then the optical follow-ups were performed using confocal laser scanning microscopy and optical coherence tomography right after laser application and during the following 3 weeks. Both of these techniques were able to visualize the therapeutical effects of the laser therapy, suggesting that noninvasive methods could be used to evaluate the efficacy of laser treatments [23].

The aim of the present study was to investigate the efficacy of the combined 585/1064 nm laser treatment of surgical scars and to evaluate the short-term effects by* in vivo* confocal microscopy and the long-term effects by VSS. Surgical scar treatment with the combined PDL/Nd:YAG laser therapy starting on the day of suture removal has not been reported previously.

In our study, one half of each of the postoperative linear scars immediately after suture removal was treated three times at monthly intervals with the combined 585/1064 nm laser and the cosmetic appearances of the treated and untreated scar halves were compared. The VSS parameters indicated a significant improvement at the final assessment relative to the control. Our results are in concordance with recent data of other authors on the topic.

When additional surgical scars (*n* = 7 scars) were similarly treated only once with the combined 585/1064 nm laser, dermatoscopy and RCM one week later demonstrated an improvement in the treated area, which exhibited only low numbers of narrow horizontal vessels and decreased amounts of collagen fibers, the orientations of which were not uniformly parallel. We examined both parts of the scar by dermatoscopy and RCM one week later since the high temperature applied induces occlusion and absorption of abnormal blood vessels in the scar tissue area, which results in artificial ischemia, inhibiting nutrient supply to the wound. We eliminated the subjectivity of the independent examiner; therefore, the evaluation of changes in vascularisation and rearrangement of collagen bundles was measured using RCM. We found that RCM was suitable for the monitoring of wound healing with or without the combined 585/1064 nm laser treatment. In conclusion, the combined PDL/Nd:YAG laser proved to be safe and effective in improving the cosmetic appearance (*n* = 39 scars) of surgical scars in patients with skin types I–IV, starting on the day of suture removal, and this improvement was confirmed at the structural level (*n* = 7) by* in vivo* confocal microscopy. RCM proved to a valuable tool for monitoring the efficacy of laser treatment of the scars.

## Figures and Tables

**Table 1:** Patients' data: 25 participants between the ages of 17 and 50 years with Fitzpatrick skin types I–IV and linear scars greater than 3 cm were enrolled into the study.

Patient	Sex	Age (years)	Scars (number of pieces)	Fitzpatrick skin types	Location
1	F	40	1	II.	Abdomen
2	F	20	1	III.	Scapula
3	F	35	1	II.	Abdomen
4	F	26	2	II.	Back
5	M	25	3	II.	Back
6	M	25	2	III.	Neck
7	M	25	1	II.	Neck
8	F	39	2	III.	Abdomen
9	F	17	1	II.	Back
10	F	23	1	I.	Waist
11	F	38	1	II.	Mons pubis
12	F	23	1	I.	Abdomen
13	F	24	1	II.	Leg
14	F	27	1	II.	Leg
15	F	30	2	III.	Back and arm
16	F	21	1	IV.	Back
17	F	30	3	II.	Neck, back, and upper arm
18	F	26	2	II.	Abdomen
19	F	25	3	II.	Abdomen
20	F	50	1	III.	Back
21	F	25	1	III.	Back
22	F	19	1	II.	Back
23	F	19	2	III.	Back
24	F	21	2	II.	Mons pubis and back
25	F	32	2	II.	Back
